# Necrotizing sarcoid granulomatosis with hemoptysis: a case report and literature review

**DOI:** 10.1186/1746-1596-8-79

**Published:** 2013-05-13

**Authors:** Haidong Huang, Chen Li, Chong Bai, Qiang Li, Weiqiang Zheng, Zhi Zhu, Paul Zarogoulidis, Konstantinos Zarogoulidis, Andreas Gschwendtner, Wolfgang Hohenforst-Schmidt, Michael Simoff

**Affiliations:** 1 Department of Respiratory Diseases, Changhai Hospital, Second Military Medical University, Shanghai, China; 2 Department of respiration, First Automobile Works General Hospital/ fourth Affiliated Hospital of Jilin University, Shanghai, China; 3 Department of Pathology, Changhai Hospital, Second Military Medical University, Shanghai, China; 4 Pulmonary Department, G. Papanikolaou`` General Hopspital, Aristotle University of Thessaloniki, Thessaloniki, Greece; 5 Department of Interventional Pneumology, Ruhrlandklinik, West German Lung Center, University Hospital, University Duisburg-Essen, Essen, Germany; 6 Pathology Department, Hospital of Amberg, Amberg, Germany; 7 II Medical Clinic, ``Coburg`` Hospital, University of Wuerzburg, Coburg, Germany; 8 Bronchoscopy and Interventional Pulmonology, Pulmonary and Critical Care Medicine, Henry Ford Hospital, Detroit, MI, USA

**Keywords:** Necrotizing sarcoid granulomatosis, Granuloma, Vasculitis, Necrosis, Bronchoscopy, Hemoptysis

## Abstract

**Virtual Slides:**

The virtual slide(s) for this article can be found here: http://www.diagnosticpathology.diagnomx.eu/vs/1955868163936338

We present a case of 39-year-old male with the symptoms of fever, cough, chest pain and bloody phlegm, whose chest CT showed multiple subpleural nodules and inflammatory infiltration. Video-Assisted Thoracic Surgery ( VATS ) for right subplural nodule was performed and confirmed the diagnosis of necrotizing sarcoid granulomatosis. Prednisolone was administered and the symptoms were under control untill the occurrence of intermittent hemoptysis after 10 months. Chest CT and bronchoscope revealed the right lower lobe nodule with intraluminal necrotic tissue in the right lower lobe posterior basal segment respectively. Fatal hemoptysis happened during endobronchial biopsy by flexible bronchoscope forcep. Based on this case, we reviewed the relevant literature and discussed the clinical features, pathological changes and prognosis of the disease.

## Background

Necrotizing Sarcoid Granulomatosis (NSG) was firstly proposed by American Pathologists Liebow in 1973, who defined 3 classical characteristics for NSG, firstly, NSG had a background of sarcoid-like granulomata histologically, a prominent and usually granulomatous vasculitis with varying degrees of necrosis; Secondly, Radiographic features with pulmonary nodules without enlarged hilar lymph nodes ; Thirdly, Most of NSG had a benign prognosis . Compared with other lung granulomatosis, NSG is an extremly rare diagnosis and can only be identified based on large pieces of pathological tissue with characteristic pathological changes such as non-caseating epithelioid cell granulomas, granulomatous vasculitis and necrosis. NSG is sensitive to corticosteroid treatment. Although it's previously considered as a disease with good prognosis, however; relapse is common. The reports of poor progrosis as well as severe complications, even death are not rare, especially wih NSG cases under long term corticosteroid control [1]. Here we report a case of NSG presenting with intermittent hemoptysis when corticosteroid therapy was decreased, and fatal hemoptysis occured during the follow-up. Relevant studies of this rare entity are also presented in the discussion section.

## Case presentation

**Figure 1:**
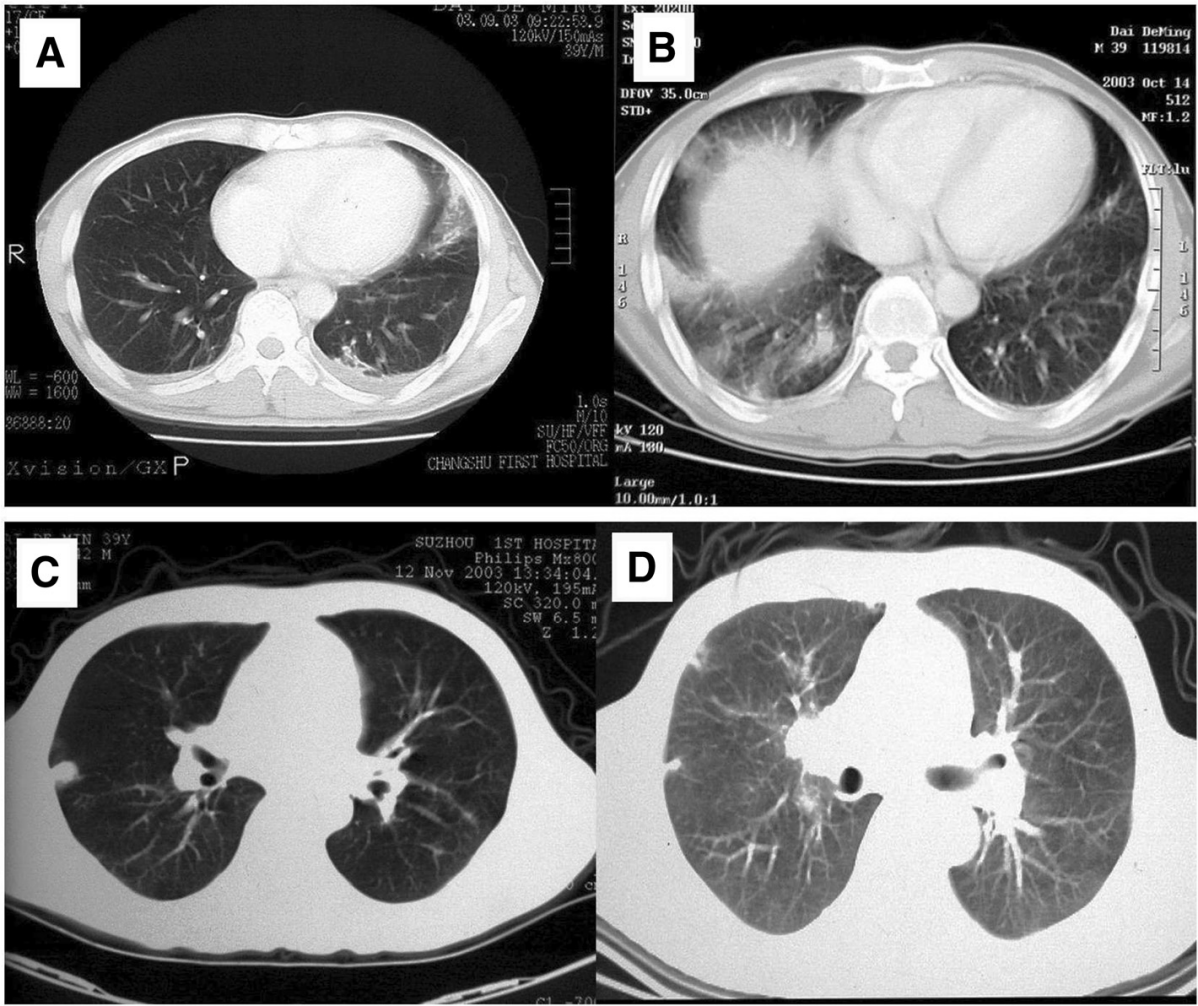
CT findings upon admission.

**Figure 2:**
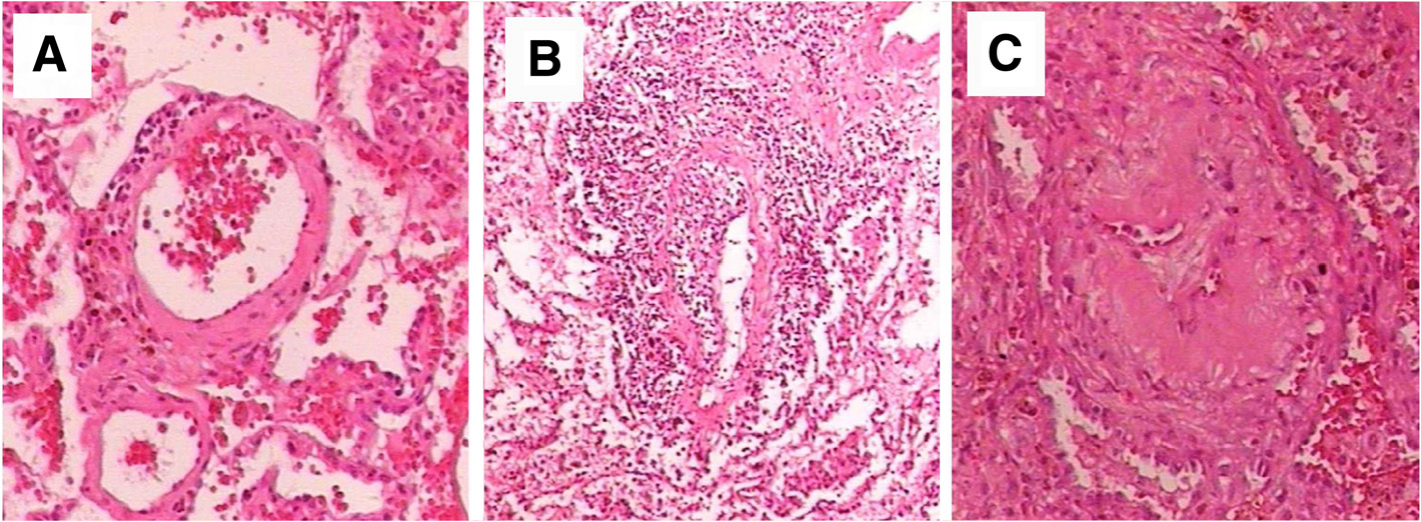
**Different degrees of small pulmonary vasculitis were showed under HE staining. A**, Vessel wall was infiltrated by lymphocytes, but the lumen is still maitain without obstruction(100×); **B**, Epithelioid granuloma invaded small vessel wall, broke into the lumen, more than 60% of the lumen was obstructed (100×); **C**, Last stage of epithelioid granulomatous vasculitis with thickening vessel wall as well as severe narrow lumen. Epithelioid granuloma invaded the vessel wall with messed vascular structures, lumen atresia (200×).

**Figure 3:**
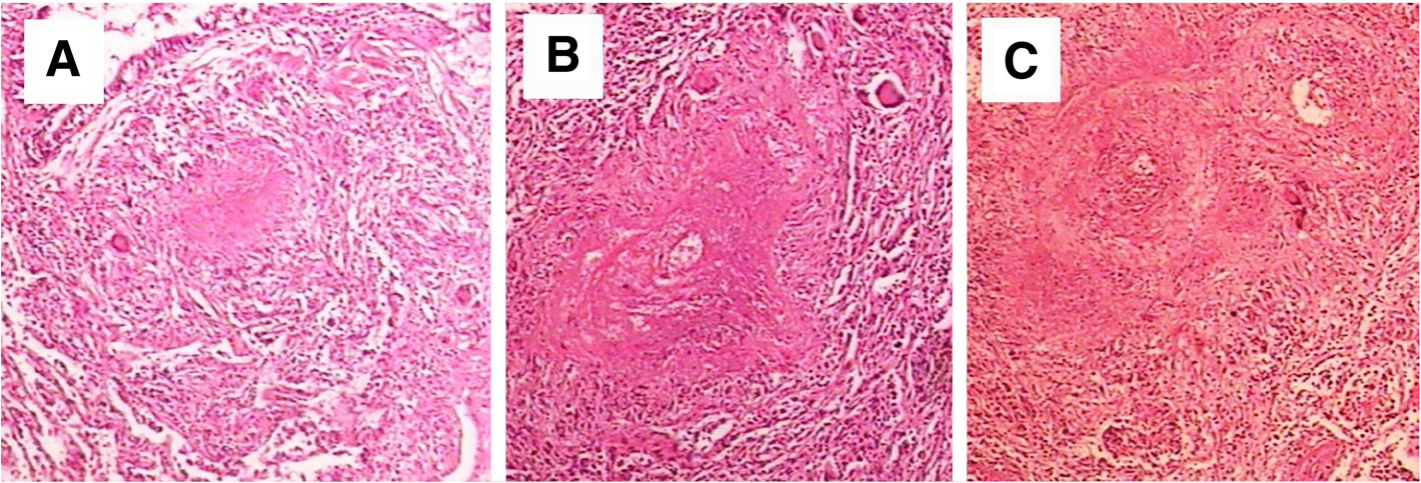
**Structures of epithelioid granuloma with varies of coagulation necrosis in the lung under HE staining. A**, Lung epithelioid granuloma with coagulation necrosis in the center, composed of a lot of lymphocytes and scattered multinucleated giant cells. Bronchial ciliated columnar epithelium structure was shown in the top left direction of figure, alveolar epithelium was shown in the lower left direction of figure (100×); **B**, Necrosis in the center of epithelioid granuloma, surrounded by lymphocytes and several multinucleated giant cells(100×);**C**, Fusion of epithelioid granuloma, amounts of coagulation necrosis located in the junction(100×).

**Figure 4:**
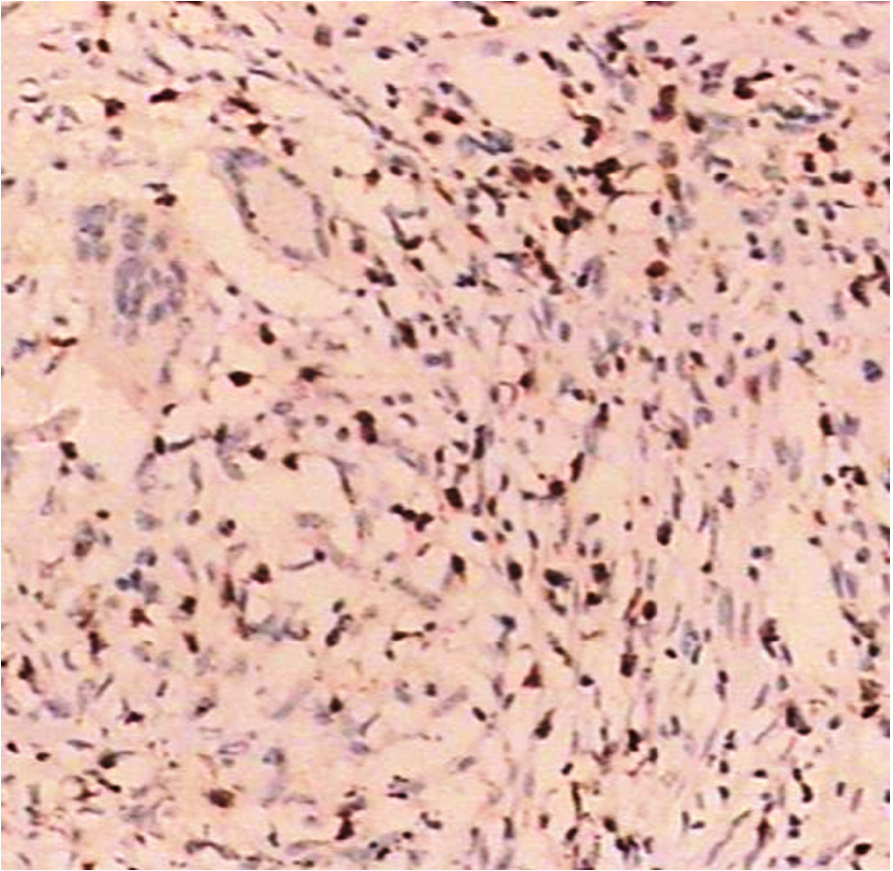
CD8+ T lymphocytes were positive with brown stained in EnVision immunohistochemistry staining (40×).

**Figure 5:**
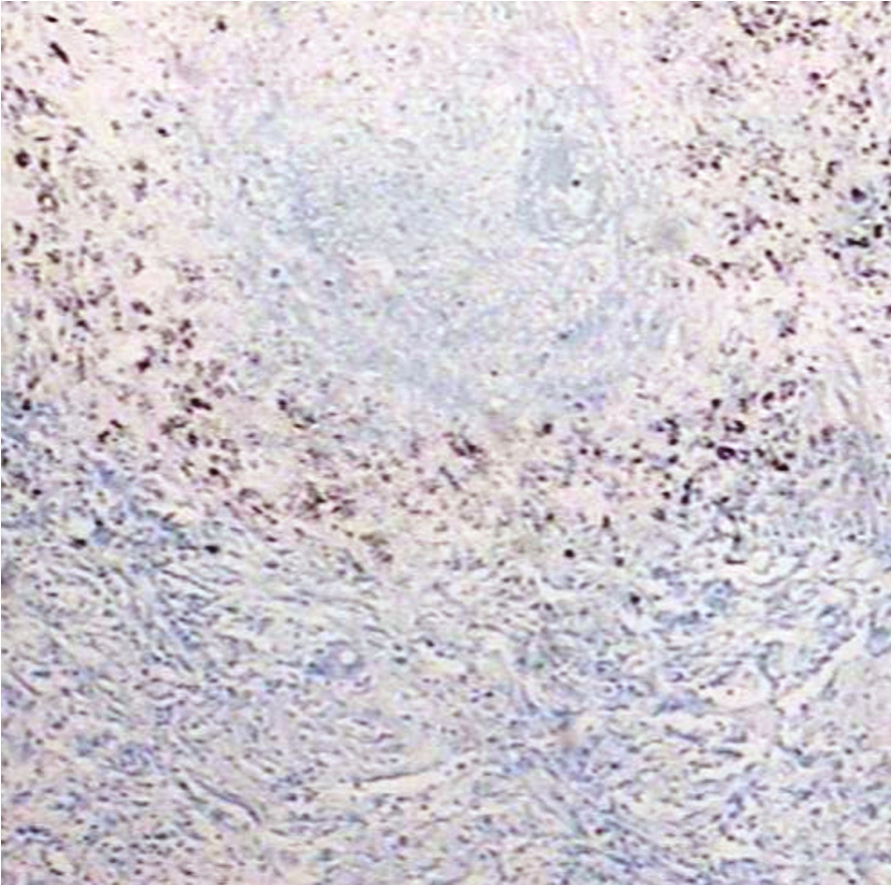
Periodic Aacid Schiff (PAS) staining was negative in a pulmonary epithelioid granuloma with coagulation necrosis (100×).

A 39-year-old male was admitted to our hospital on 27,Nov,2003 due to fever , chest pain and dry cough for 3 months. The patient had left chest pain accompanied with chills and fever since 4,Sep,2003, temperature increased after noon and reached at 39°C; high fever was accompanied with fatigue, sweating and the temperature dropped to the normal in the next morning automatically. The patient’s symtoms had no improvement and after 40 days diagnostic anti-TB treatment with isoniazid, pyrazinamide, and rifampicin in outside facility was administered. The Chest CT revealed ground-glass exudation with patchy infiltrates at the lingula lobe of the left lung and mild bilateral effusions (Figure 1A). Cephradine intravenous injection 2.0 g twice a day combined with azithromycin intravenous injection 0.5g once a day and isepamicin sulfate 0.4 once a day were administered to the patient, no symtom improvement was observed after 10days while the right chest discomfort and shortness of breath continued (Figure 1B) favoring a clinical diagnosis of TB. Administering of isoniazid, rifampicin and pyrazinamide for 30d did not relieve the symptoms with the manifestations: dry cough, anorexia and weight loss of about 10 kg. Chest CT showed improvement of the lower right lung inflammation and subpleural small nodular shadows at the right lung (Figure 1C). Upon admission at physical examination the patient was alert and oriented X3, temperature at 38°C, reduced breath sounds at the right lower lung, diminished fremitus, percussion dullness, no crackles and wheezing bilaterally on chest physical examination. Laboratory tests showed: Blood cell count: WBC (10.5×10^9^/L), neutrophils (0.73), lymphocytes (0.17) hemoglobin: (107 g/L), platelet (215×10^9^/L), erythrocyte sedimentation rate (115 mm/1h), negative results in tuberculin test and acid-fast bacilli test using multiple blood cultures and sputum. Tests of serum AFP:30(reference value <30 ug/l), CEA:1.01(reference value <4.6 ng/ml), CA-199:3.64(reference value <37  U/ml), NSE:38.81(reference value <15.2 ng/ml), PSA:0.66(reference value <4.5  ng/ml), Anti-O:76.5(reference value <250Todd), RF:10.3(reference value <20 IU/ml), serum P-ANCA, C-ANCA, HIV were negative. pulmonary function test showed significantly decreased pulmonary ventilation function, normal pulmonary gas exchange function and normal small airway examination. Flexible bronchoscope was performed with normal bronchoscopic airway findings on 2,Dec,2003. Conventional transbronchial needle aspiration biopsy was performed on the site of sub-carina lymph nodes which was negative of malignant cell. Endobronchial brush was done in the distal of lateral segment of right middle lobe, which was negtive for malignant cell as well as acid-fast bacilli. Electric Bronchoscope equipment including bronchoscope type with OLYMPUS BF P240, light source with OLYMPUS CV-240, Image processor with OLYMPUS CLV-U40 were used during the procedure. In order to clarify the diagnosis, surgical biopsy was considered. Before the procedure, chest CT was performed again to determine the biopsy site, which showed small subpleural nodular shadows at the right upper lobe as well as enlarged right hilar (Figure 1D). Subpleural nodules of RUL was obtained by VATS and sent for pathology examination, which showed gray nodules with clear boundary during surgery. Microscopic analysis demonstrated scattered epithelioid cell granuloma with central coagulation necrosis surrounded by the multinucleated giant cells; destruction of small pulmonary arteries or small veins and infiltration of lymphocytes, monocytes and plasma cell; nodules surrounded by fibrous tissue and necrotizing vasculitis and infiltration of vascular parietal lymphocytes and hemosiderin cells; occlusion of the vessel lumen (Figures 2 and 3). CD8+T-lymphocytes were positive with brown stained in EnVision immunohistochemistry staining (Figure 4). Acid-fast staining and PAS staining showed negative results (Figure 5). The final pathological diagnosis was NSG. Equipment with Olympus U-SPT microscope and Sony Digital Hyper HAD Colour Video Camera were used by pathologists for tissue observation and imaging.

**Figure 6:**
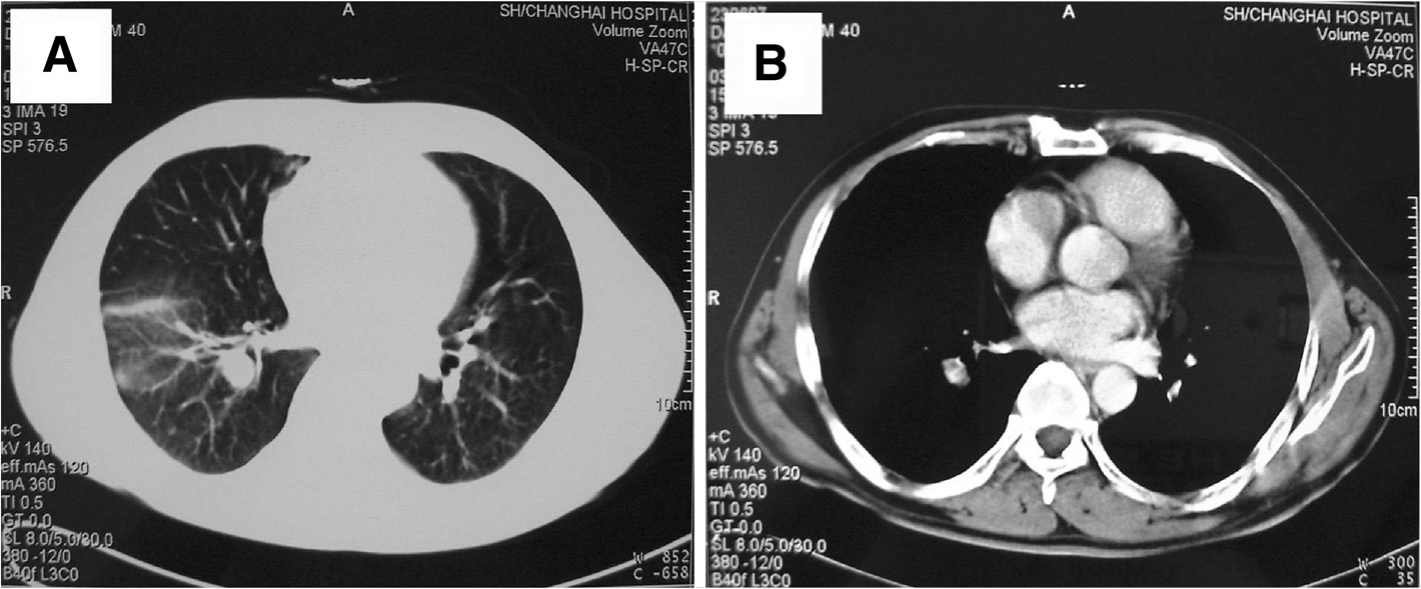
CT findings upon follow up.

**Figure 7:**
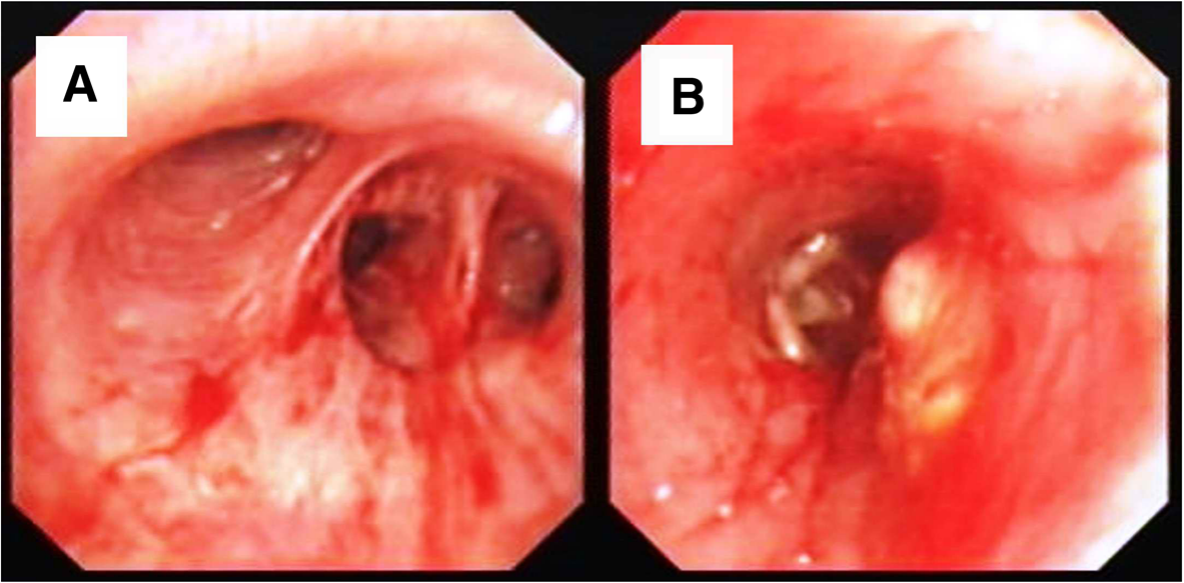
Bronchoscopy findings for biopsy.

The patient tolerated the surgical procedure well and temperature recovered the next day after VATS, Prednisone tablet 20mg three times a day oral was given 5d after the surgery for 3 days, then reduced to 15mg three times a day oral for 5 days, then keep reducing to 10mg three times a day oral for 5 days. Patient did well with relieved fever and chest discomfort. We therefore gradually reduced the dosage of prednisone and maintained prednisone tablet 10 mg/d since the 6^th^ month after VATS. Patient's temperature remained normal and general conditions improved completely. However, hemoptysis (100–200 ml/d) occurred to the patient after the emotion or exertion on the 10^th^ month after VATS. The symptoms persisted with low fever and the spit of purple-black blood clots could be observed. Chest contrast CT in December 2004 showed soft tissue shadow at the basal segment of the right lower lobe, (Figure 6). In order to role out tuberculosis and other infection diseases, Flexible Bronchoscope was performed again, which showed mild bleeding coming from the posterior basal segment of right lower lobe with intraluminal yellow-white necrotic tissue (Figure 7). Massive bleeding occurred suddenly at the third times forcep endobronchial biopsy of intraluminal necrotic tissue Emergency rescue measures including right lateral position, intravenous use of pituitrin and intubation were performed immediately, but failed to control the fatal bleeding. The patient eventually passed away due to asphyxiation by the complete obstructing of airway blood clots after 120 miniutes CPR procedure.

## Conclusions

NSG is an extremely rare granulomatosis, only about 200 cases were reported in English literature in pubmed. Its etiology and pathogenesis still remains unclear. Some scholars believed that some kind of infection, such as aspergillus, chlamydia pneumoniae and immune disorder mechanism post infection involved in the pathogenesis of the NSG [2-4]. Heinrich et al. [5] firstly reported that reduced peripheral blood CD4/CD8 T cell ratio and increased CD4/CD8 T cell ratio at the lesions indicating immune dysfunction is involved in the pathogenesis. The antigen-antibody complexes eventually induce immune disorders and disease. Compared to the other organs, lung is rich with blood supply and the deposit of antigen-antibody is common, which makes lung the most frequently involved organ for NSG. This could also explain the occurrence of NSG associated with various autoimmune diseases, such as biliary cirrhosis, ankylosing spondylitis and rheumatoid arthritis [6,7].

The average age for the onset of NSG is between 40–60 years and is dominant in female. NSG's clinical manifestations are normally mild and unspecific such as chest pain (63%), cough (38%), fever (30%) and general malaise (31%). In addition to the lung, multiple reports showed NSG is accompanied with skin erythema nodosum, uveitis, and involvement of the stomach, liver and central nervous system [2,5,6,8-14].

In the present case, clinical manifestations such as recurrent fever and chest pain were consistent with previous reports. The current case also showed characteristics of NSG image: solitary or multiple nodules or lumps shadow of lung associated with local pleural thickening in chest radiography; Chest CT showed visible pulmonary nodules distributed subpleurally or along the bronchi and some nodules were empty. Other reported clinical manifestations included hilar lymphadenopathy (28%), pleural effusion (11%) and nodular lesions within the vacuoles (8%) [5,6,8,15,16]. NSG also shares the similarities of the migratory and varied lesions with disease like pulmonary vasculitis [17]. It was reported that positron emission computed tomography (F-18FDG / PET-CT) had been used to check NSG patients, which was able to show the actual NSG extent, detect the SUV value of NSG lesions and guide the surgical biopsy at the same time [18].

Diagnosis of NSG mainly relies on pathological findings, non-caseating epithelial cell granulomas, granulomatous vasculitis and necrosis which are the main pathological features of NSG. Chronic granulomatosis caused by tuberculosis, atypical mycobacteria, fungi and parasites should be excluded for differential diagnosis. Non-caseating epithelial cell granulomas of NSG may invade the vessel wall and lumen to form granulomatous vasculitis and lesions of coagulation necrosis; lymphocytes, multinucleated giant cells and other inflammatory cells can infiltrate pulmonary blood vessel walls and cause severe stenosis or occlusion of the vessel lumen with perivascular granuloma formation [1,6,11,16,19]. The granulomatous vasculitis can lead to the ischemic necrosis surrounding the blood vessels, a unique pathological feature of NSG. In severe cases, it can cause extensive intrapulmonary coagulation necrosis and intrapulmonary hemorrhage, alveolar bleeding and widely distributed hemosiderin cells [7,11,20-23].

In the present case severe granuloma and necrosis at the lung nodules was observed. In consistent with the previous reports, microscopic observation showed the severe pathological vascular inflammation and necrosis (Figures 2 and 3). It's arguably believed that NSG and sarcoidosis are the same disease since NSG has pathological features of both sarcoidosis and vasculitis , they share similar non-caseating epithelial cell granulomas [2,20,24]. However, NSG does has distinct clinical features from sarcoidosis such as both negative serum angiotensin converting enzyme (SACE) and Kveim test. Moreover, no lung hilar and mediastinal lymph nodes are present in NSG patients and no asteroid bodies and Schaumann bodies, which are often found in sarcoidosis patients [25]; Granulomatous vasculitis and coagulation necrosis of NSG are generally more severe than sarcoidosis. NSG laboratory findings are also different from the systemic vasculitis (such as: Wegener granulomatosis) where the serum C-ANCA, P-ANCA are negative in NSG patients, and the granulomatous type and prognosis of NSZZG are also different. Although there are some same identical points on clinical manifestation between NSG and limited Wegener’s disease, such as either negative serum ANCA or lacking of evidence of glomerulonephritis, but actually they are completely different diseases, especially in pathological findings. The main pathological features of limited Wegeners’ in the lung including liquefaction and coagulation necrosis lesions, as well as a large number of eosinophils, and a small amount of lymphoplasmacytic infiltration, the destructive vasculitis can be found in the small pulmonary arteries and small veins. Comparing to this case, although a large number of necrosis and vasculitis could be found in the lesion, but not like NSG, the necrosis form in this case presented more like infarction necrosis post vascular obstruction due to the granulomatosis vasculitis, there was also no significant liquefaction in necrotic lesion. The inflammation cells in this case mainly composed of lynphocyte cells, rather than eosinophils cells. In this case, although there were different degrees of vascular inflammation and thickening of the vessel wall, most of the vascular wall structure still existed, rather than the destructive vasculitis such as in limited Wegners’ disease. In general, according to the pathological features in microscope of this case, we believed that this was a classical NSG case [9].

NSG is sensitive to corticosteroid treatment but relapse is not rare [11,19]. Although NSG is considered as a disease with good prognosis generally, the reported cases with poor prognosis are increasingly common with in-depth understanding of the disease as well as the extended follow-up period. In a multi-center retrospective study of 14 cases with follow-up time of 18–114 months, 50% of patients demonstrated poor prognosis: 1 patient died of central nervous system infection; two cases presented lung cancer in long-term follow-up; 4 cases has recurrence [9]. Other reports also presented severe complications with long term maintenance therapy of corticosteroids such, such as fungal infections, lung tumor and even death [2,6-8,26]. Therefore, clinical management requires particular attention to the NSG cases under long term long term corticosteroid treatment. Once the symptoms get worse with recent solitary nodule or cavitary lesion, further examination including biopsy is required.

In the present case, low-dose oral corticosteroid therapy was effective but hemoptysis with fever occurred during gradual reduction of corticosteroids with the image of the lower right hilar neoplasm. We considered several possibilities such as 1) NSG relapse and lung granuloma invading the vessel wall resulting in bleeding; 2) secondary pulmonary infection, such as tuberculosis, or fungal infection; 3) secondary lung tumor. Endobronchoial biopsy by flexible bronchoscope forcep normally is considered the routine approach for differential diagnosis, however unexpectedly fatal hemoptysis happened after biopsy and led to death, a rare complication which was not present in previous NSG reports. Based on the available literature, biopsy forceps clamp caused hemoptysis after bronchoscopy is common in bronchial dieulafoy disease [27,28]. However, the patient had no past medical history of hemoptysis before, the previous chest CT scan and flexible bronchoscopy exam also did not show the basal segment abnormalities at the right lower lobe. Given the severe epithelial cells granulomatous vasculitis and extensive necrotic lesions in the pathological findings of VATS, we speculated NSG relapsed during the reduction of corticosteroid treatment. The recurrence site located in the lower right lung nearly hilar and severe granulomatous vasculitis and necrosis invaded the bronchial lumen of the basal segment as well as adjacent artery leading to the formation of bronchovascular fistula, and endobronchial biopsy procedure with mechanical force caused further damage to the bronchus and blood vessel wall. Previously two reports also showed complications of hemoptysis in the long-term follow-up process of NSG [9,29]. After reviewing of enhanced chest CT images, we could observe a clear contrast agent enhancement at basal segment of the right lower lobe suggesting NSG granuloma may invade the blood vessels of basal segments at the right lower lung. Necrosis-like lesions caused by infection or tumor unlikely would cause such massive and rapid bleeding during biopsy. Unfortunately, the patient’s family refused autopsy. In one case study, serum interleukin-2 receptor restored to normal level with the improved condition and imaging results suggesting the value of interleukin-2 in NSG's prognosis [30]. There is still no clear association of the severity of granulomatous vasculitis and necrosis in correlation to NSG prognosis. However, it was observed that the cases with less severe NSG granulomatous vasculitis or necrosis had better prognosis [7]. In the present case the patient with severe lung granulomatous vasculitis and necrosis had rapid clinical relapse and lethal hemoptysis.

We firstly reported the NSG case with the fatal complication of massive hemoptysis during the flexible bronchoscope endobronchial biopsy. In particular, special attention should be paid to the patients with necrosis-like lesions during bronchoscopic biopsy to avoid lethal hemoptysis. Based on the clinical course and pathological features of our case, we presented the clinical conjecture that NSG cases with severe granulomatous vasculitis and necrosis in pathology would have a poor prognosis. So far, it's still lack of comprehensive and in-depth studies on this disease. Further studies on the etiology and pathogenesis of NSG will pave the way to the understanding of the classification, diagnosis, treatment and prognosis of NSG.

## Consent

Written informed consent was obtained from the patients next of kin for publication of this Case report and any accompanying images. A copy of the written consent is available for review by the Editor of this journal.

## Abbreviations

NSG: Necrotizing sarcoid granulomatosis; SACE: Serum angiotensin converting enzyme; PSA: Prostate-specific antigen; RF: Rheumatoid factor; P-ANCA: Perinuclear Anti-Neutrophil Cytoplasmic Antibodies; C-ANCA: Cytoplasmic antineutrophil cytoplasmic antibodies; HIV: Human immunodeficiency virus; CT: Computed Tomography; PET-CT: Positron Emission Tomography - Computed Tomography; AFP: Alpha-Fetal Protein; CEA: Carcinoembryonic antigen; CA-19.9: Carbohydrate antigen.

## Competing interests

The authors declare that they have no competing interest.

## Authors’ contributions

HH and CL diagnosed and treated the patient. HH and CL contributed equally to the manuscript. CB and WZ performed the bronchoscopy. QL is the director of the department and provided useful insights. PZ, HH and CL wrote and prepared the manuscript. AG assisted in the evaluation as an external pathologist. KZ, MS and WH-S assisted as external physician useful insights. All authors read and approved the final manuscript.
